# 

**Published:** 1921-05-14

**Authors:** 


May 14, 1921] [Price Twopence

### The Hospital

The Workers' Newspaper of Administrative Medicine and Institutional Life, Administration, National Insurance and Health.

CONTENTS

Medical Representation ... ... ... ... 107
The Hospital Pageant ... ... ... ... ... 107
The Editor's Letter-Box ... ... ... ... 108
Is a Hospital Pageant Practicable?—The Royal Hospital for Incurables.
Notes of the Week ... ... ... ... ... 109
The New Minister and the British Medical Association—The B.M.A. Meeting—Approved Societies and Hospitals—Drastic Reductions at the London Hospital—A Super-Nursing Home—Coal Permits for Hospitals—An Unwanted Laboratory—Tonsils and Adenoids—The Prince's Portrait—Hospitals for the Middle Class—Hospital Horticulture in Sussex—Retirement of Dr. Bedford Pierce—The Effect of Closing Beds.
Science and Eye Strain ... ... ... ... 111
The Effects of Invisible Solar Rays.
The Institutional Library .. ... ... 113
The Right to Strike—Blood Pictures: An introduction to Clinical Hæmatology—Heart Disease: Graphic Methods—Care and Feeding of Infants and Children—Model Answers to Questions on Nursing, set by the Medico-Psychological Association—Bio-Moulding Origins in Evolution—First-Aid in Emergencies.
Annual Meetings of Hospitals ... ... ... 114
Passing Events and Topics ... ... ... ... 114
The Public Well-Being ... ... ... ... ... 115
The "Newman Policy"—A "Revolting" Crime in Scotland—The Guardianship of Children—The Fly Pest.
The Matrons' and Sisters' Department ... ... 117
"Qualified Nurses" and their Qualifications. One Portal—with a Difference.
Round the Hospitals ... ... ... . ... 118
Matrons' and Sisters' Appointments ... ... 119
Nursing Authorities in Conference ... ... 120
Alternative and Reciprocal Training.
The British Hospitals Association ... ... ... 121
Parliament and the Profession ... ... ... 121
The Draft Syllabus: Official Explanation ... 122
The National Children Adoption Association ... 122
The College of Nursing (Limited by Guarantee) vi

